# The Antibacterial Efficacy of High-Fluence PACK Cross-Linking Can Be Accelerated

**DOI:** 10.1167/tvst.12.2.12

**Published:** 2023-02-09

**Authors:** Nan-Ji Lu, Hendrik Koliwer-Brandl, Francesca Gilardoni, Nikki Hafezi, Boris Knyazer, Asaf Achiron, Reinhard Zbinden, Adrian Egli, Farhad Hafezi

**Affiliations:** 1 School of Ophthalmology and Optometry, Wenzhou Medical University, Wenzhou, Zhejiang, China; 2 School of Medicine and Health Sciences, University of Antwerp, Wilrijk, Belgium; 3 ELZA Institute, Dietikon, Zurich, Switzerland; 4 Institute of Medical Microbiology, University of Zurich, Zurich, Switzerland; 5 Department of Ophthalmology, Soroka University Medical Center, Faculty of Health Sciences, Ben-Gurion University of the Negev, Beer-Sheva, Israel; 6 Tel Aviv Sourasky Medical Center and Sackler School of Medicine, Tel Aviv University, Tel Aviv, Israel; 7 Ocular Cell Biology Group, Center for Applied Biotechnology and Molecular Medicine, University of Zurich, Zurich, Switzerland; 8 USC Roski Eye Institute, University of Southern California, Los Angeles, CA, USA; 9 Faculty of Medicine, University of Geneva, Geneva, Switzerland

**Keywords:** corneal cross-linking, keratitis, antibacterial efficacy, bacterial strain

## Abstract

**Purpose:**

To determine whether high-fluence photoactivated chromophore for keratitis cross-linking (PACK-CXL) can be accelerated.

**Methods:**

Solutions of *Staphylococcus aureus* and *Pseudomonas aeruginosa* with 0.1% riboflavin were prepared and exposed to 365 nm ultraviolet (UV)-A irradiation of intensities and fluences from 9 to 30 mW/cm^2^ and from 5.4 to 15.0 J/cm^2^, respectively, representing nine different accelerated PACK-CXL protocols. Irradiated solutions and unirradiated controls were diluted, plated, and inoculated on agar plates so that the bacterial killing ratios (BKR) could be calculated. Additionally, strains of *Achromobacter xylosoxidans, Staphylococcus epidermidis*, and *Stenotrophomonas maltophilia* were exposed to a single accelerated PACK-CXL protocol (intensity: 30 mW/cm^2^, total fluence: 15.0 J/cm^2^).

**Results:**

With total fluences of 5.4, 10.0, and 15.0 J/cm^2^, the range of mean BKR for *S. aureus* was 45.78% to 50.91%, 84.13% to 88.16%, and 97.50% to 99.90%, respectively; the mean BKR for *P. aeruginosa* was 69.09% to 70.86%, 75.37% to 77.93%, and 82.27% to 91.44%, respectively. The mean BKR was 41.97% for *A. xylosoxidans*, 65.38% for *S. epidermidis*, and 78.04% for *S. maltophilia* for the accelerated PACK-CXL protocol (30 mW/cm^2^, 15 J/cm^2^).

**Conclusions:**

The BKR of high-fluence PACK-CXL protocols can be accelerated while maintaining a high, but species-dependent, BKR. The Bunsen to Roscoe law is respected in fluences up to 10 J/cm^2^ in *S. aureus* and *P. aeruginosa*, whereas fluences above 10 J/cm^2^ show strain dependence.

**Translational Relevance:**

The high-fluence PACK-CXL protocols can be accelerated in clinical practice while maintaining high levels of BKR.

## Introduction

Infectious keratitis is a significant and frequent cause of ocular morbidity.^1^^,^^2^ Typically, the infection requires urgent and repeated treatment, which involves the intense and continuous application of antimicrobial agents.^3^^,^^4^ Most cases are bacterial, fungal, or mixed (bacterial/fungal) in origin; however, the timely identification of the causative organism(s) and selection of the most appropriate antimicrobial agent(s) sometimes can be challenging.^5^ Unfortunately, increasing antimicrobial resistance continues to decrease the number of effective treatment options, leading to a growing need for new infectious keratitis treatments to overcome the challenge of antimicrobial resistance. Photoactivated chromophore for keratitis-corneal cross-linking (PACK-CXL) represents one such approach.^6^

Corneal CXL, originally developed for the treatment of corneal ectasia, involves the saturation of the cornea with a chromophore, which is then photoactivated in situ. Typically, the chromophore is riboflavin, which is photoactivated with 365-nm ultraviolet-A (UV-A) light, generating riboflavin radicals and reactive oxygen species. The latter has multiple effects: (1) the covalent bonds between collagen molecules and proteoglycans of the extracellular matrix increase biomechanical stiffness, (2) increased steric hindrance and altered access to metalloproteinase cleavage sites render the corneal stroma more resistant to enzymatic digestion, and (3) increased oxidative stress induces a direct damaging effect on the cell membranes and nucleic acids of any bacterial and fungal pathogens present.^7^ PACK-CXL has been successfully applied for killing bacteria and fungi in experiments; it has also been shown to be effective alone and in combination with standard-of-care antimicrobial therapy in clinical practices.^8^^–^^12^

We have previously shown in vitro that high-fluence PACK-CXL distinctly increases the bacterial killing ratio (BKR).^13^ The purpose of this study was to determine whether high-fluence PACK-CXL can be accelerated while maintaining its antibacterial efficacy, because accelerated high-fluence protocols are not only more time saving, but also more suitable to be applied at the slit lamp, giving more convenient access to the treatments and a better and more comfortable treatment experience for patients.

## Methods

### Bacterial Strains and UV-A Light Device

Bacterial strains are independent clinical isolates of corneal infections: *Staphylococcus aureus* (methicillin-sensitive), *Pseudomonas aeruginosa*, *Achromobacter xylosoxidans*, *Staphylococcus epidermidis*, and *Stenotrophomonas maltophilia*, isolated at the Institute of Medical Microbiology, University of Zurich. The same portable 365-nm UV light source with a fixed 12-mm irradiation spot that covered each single 96 empty plate aperture (C-Eye; EMAGine AG, Zug, Switzerland), was used to perform all UV-A irradiations.

### Preparation of Bacterial Solutions

Colonies of all bacterial strains from overnight subcultures on Colombia agar + 5% sheep blood (bioMérieux, Marcy l'Etoile, France) were suspended in sterile 0.9% NaCl and adjusted to McFarland 0.5, corresponding to approximately 10^8^ colony-forming units (CFU)/mL, in the tubes. Then, the bacterial solutions were diluted 10-fold to 10^7^ CFU/mL in the 96-well microtiter plates (Costar Assay Plate, Corning Incorporated, NY) that contained a final concentration of 0.1% (w/v) riboflavin (hypo-osmolar 0.1% riboflavin without carriers, Ribo-Ker, EMAGine, Zug, Switzerland) or not to generate two kinds of standard samples, which were named as control-blank and control-riboflavin, respectively.

### Design of PACK-CXL Protocols

Nine PACK-CXL protocols, named from protocol 1 to protocol 9, were established. As shown in Table 1, three different total fluences were set, including 5.4, 10.0, and 15.0 J/cm^2^; these fluences were reached by variable UV-A intensities (9, 18, and 30 mW/cm^2^, respectively). All protocols were tested for *S. aureus* and *P. aeruginosa*, although only protocol 9 was tested for the remaining three bacterial strains, *A. xylosoxidans*, *S. epidermidis*, and *S. maltophilia*.

**Table 1.** The Technical Details of Each PACK-CXL Protocol

Protocols	Total Fluence (J/cm^2^)	Irradiance Intensity (mW/cm^2^)	Irradiance Time (mm′ ss″)
Protocol 1	5.4	9	10’00’’
Protocol 2	5.4	18	5’00’’
Protocol 3	5.4	30	3’00’’
Protocol 4	10.0	9	18’31’’
Protocol 5	10.0	18	9’15’’
Protocol 6	10.0	30	5’33’’
Protocol 7	15.0	9	27’46’’
Protocol 8	15.0	18	13’53’’
Protocol 9	15.0	30	8’20’’

mm = minutes; ss = seconds.

### Experimental Procedures and Group Settings

For all bacterial strains, 11 µL of the two standard samples (control-blank and control-riboflavin) were transferred into two separate new 96-well microtiter plates and either exposed to the experimental PACK-CXL protocols or left unirradiated. Accordingly, four study groups were generated for all bacterial strains: (1) group A: control-blank without PACK-CXL; (2) group B: control-riboflavin without PACK-CXL; (3) group C: control-blank with PACK-CXL; and (4) group D: control-riboflavin with PACK-CXL. For groups C and D, based on the PACK-CXL protocols, various protocols were applied correspondingly for all bacterial strains.

After performing PACK-CXL or not, the suspensions of all groups were 10-fold diluted three times to a concentration of approximately 10^5^ CFU/mL. Ten microliters of the final dilution were then plated on the Colombia agar + 5% sheep blood and incubated at 37°C for 24 hours to determine the bacterial reduction after irradiation. To address both biological and experimental variability to obtain reliable and stable results, all experiments were repeated using multiple independent bacterial solutions, each irradiation was repeated three times (technical replicate), and the whole experiment was repeated on three different days (biological replicate), resulting in a total of nine irradiations.

### Bacterial CFUs: Counting and Analysis

After incubation, all agar plates were photographed and the number of CFUs was counted. Because the load of the prepared bacterial solutions was set to McFarland 0.5, corresponding with 10^8^ CFU/mL for most bacteria, when 10 µL of the final resulting dilutions were plated, this meant that the untreated bacterial solutions (group A and group B) reached approximately 10^3^ CFU/10 µL on agar plates.

As described,^13^ the BKR was calculated by comparing the CFU of each PACK-CXL irradiated plate (CFU _With PACK-CXL_) with its corresponding control plate (CFU _Without PACK-CXL_) (group B vs. group D), using the following formula:

$$\begin{array}{c} BKR=\left(1-\frac{CFU_{\;With\;PACK-CXL}}{CFU_{\;Without\;PACK-CXL}}\right)\times 100\left[\%\right]. \end{array}$$

The BKRs of all bacteria strains were then compared under the same series of PACK-CXL protocols.

### Statistical Analysis

Statistical analysis was conducted using SPSS version 24 (IBM Corp., Armonk, NY) and the graphs were created in R software (version 4.2.0, The R Foundation for Statistical Computing, Vienna, Austria). A Shapiro–Wilk test was applied to verify the normality of data distribution. Descriptive statistics were described as mean ± standard deviation. Either a one-way analysis of variance or a Kruskal–Wallis H test was conducted for continuous variables to analyze the equivalence among all groups, and post hoc tests were performed with Bonferroni correction. A *P* value of less than 0.05 was considered as the threshold for statistical significance.

## Results

### Quantification of Bacteria

All five strains in groups A, B, C, and D were repeated nine times with stable results and included in the analyses. The repetitions include three biological replicates on independent days, each with three technical triplicates. For group A (control-blank without PACK-CXL), the mean CFU/10 µL were 945.22 ± 98.83, 965.11 ± 36.69, 994.22 ± 30.48, 277.11 ± 22.42, and 915.44 ± 55.46 in *S. aureus*, *P. aeruginosa*, *A. xylosoxidans*, *S. epidermidis*, and *S. maltophilia*, respectively. For group B (control-riboflavin without PACK-CXL), the average CFU/10 µL were 932.78 ± 64.70, 956.22 ± 76.66, 979.33 ± 18.23, 282.44 ± 29.81, and 932.11 ± 51.94 in *S. aureus*, *P. aeruginosa*, *A. xylosoxidans*, *S. epidermidis*, and *S. maltophilia*, respectively. There was no significant difference between group A and group B for all bacterial strains (all *P* > 0.05), meaning riboflavin itself has no significant bactericidal activity.

For group C (control-blank with PACK-CXL; without riboflavin) and group D (control-riboflavin with PACK-CXL), the average CFU/10 µL and comparisons of *S. aureus* and *P. aeruginosa* for all protocols are shown in Table 2. For group C, the average CFU/10 µL was 825.89 ± 37.54, 230.67 ± 20.83, and 612.44 ± 21.87 in *A. xylosoxidans*, *S. epidermidis*, and *S. maltophilia*, respectively; for group D, the average CFU/10 µL was 568.44 ± 43.66, 97.78 ± 16.80, and 204.67 ± 10.39 in *A. xylosoxidans*, *S. epidermidis*, and *S. maltophilia*, respectively. Statistical differences were found between group C and group D in *A. xylosoxidans*, *S. epidermidis*, and *S. maltophilia* (all *P* < 0.001), presenting that the bactericidal activity was enhanced by the addition of riboflavin (group D) compared to the UV-A light irradiation (group C).

**Table 2.** Quantification of Bacteria in CFU Before and After Different PACK-CXL Protocols

	*S. Aureus*	*P. Aeruginosa*	*S. Aureus*	*P. Aeruginosa*	*S. Aureus*	*P. Aeruginosa*
	PACK-CXL protocol 1		PACK-CXL protocol 4		PACK-CXL protocol 7	
Group C	787.33 ± 16.34	333.44 ± 37.72	703.44 ± 20.46	252.78 ± 23.69	360.33 ± 39.30	172.67 ± 16.01
Group D	505.78 ± 36.51	279.44 ± 56.29	148.00 ± 39.31	207.44 ± 35.25	0.89 ± 0.74	78.44 ± 15.99
*P* value	<0.001	0.038	<0.001	0.007	<0.001	<0.001
	PACK-CXL protocol 2		PACK-CXL protocol 5		PACK-CXL protocol 8	
Group C	779.56 ± 28.82	346.78 ± 32.06	533.67 ± 46.06	259.56 ± 44.41	290.56 ± 45.01	223.22 ± 58.43
Group D	464.56 ± 60.45	283.22 ± 36.16	110.44 ± 26.31	202.22 ± 11.33	26.00 ± 19.92	131.11 ± 23.43
*P* value	<0.001	0.002	<0.001	0.003	<0.001	<0.001
	PACK-CXL protocol 3		PACK-CXL protocol 6		PACK-CXL protocol 9	
Group C	764.22 ± 35.10	346.33 ± 68.35	529.89 ± 97.66	275.11 ± 34.05	366.78 ± 46.97	239.78 ± 35.40
Group D	457.89 ± 39.41	267.00 ± 32.12	140.78 ± 43.19	225.67 ± 10.95	23.33 ± 7.26	162.44 ± 9.65
*P* value	<0.001	0.007	<0.001	0.001	<0.001	<0.001

After PACK-CXL, the bacterial solutions were diluted to reach at the maximum approximately 10^3^ CFU on agar for CFU determination.

### BKRs in *S. Aureus* and *P. Aeruginosa*

**Figure 1.**
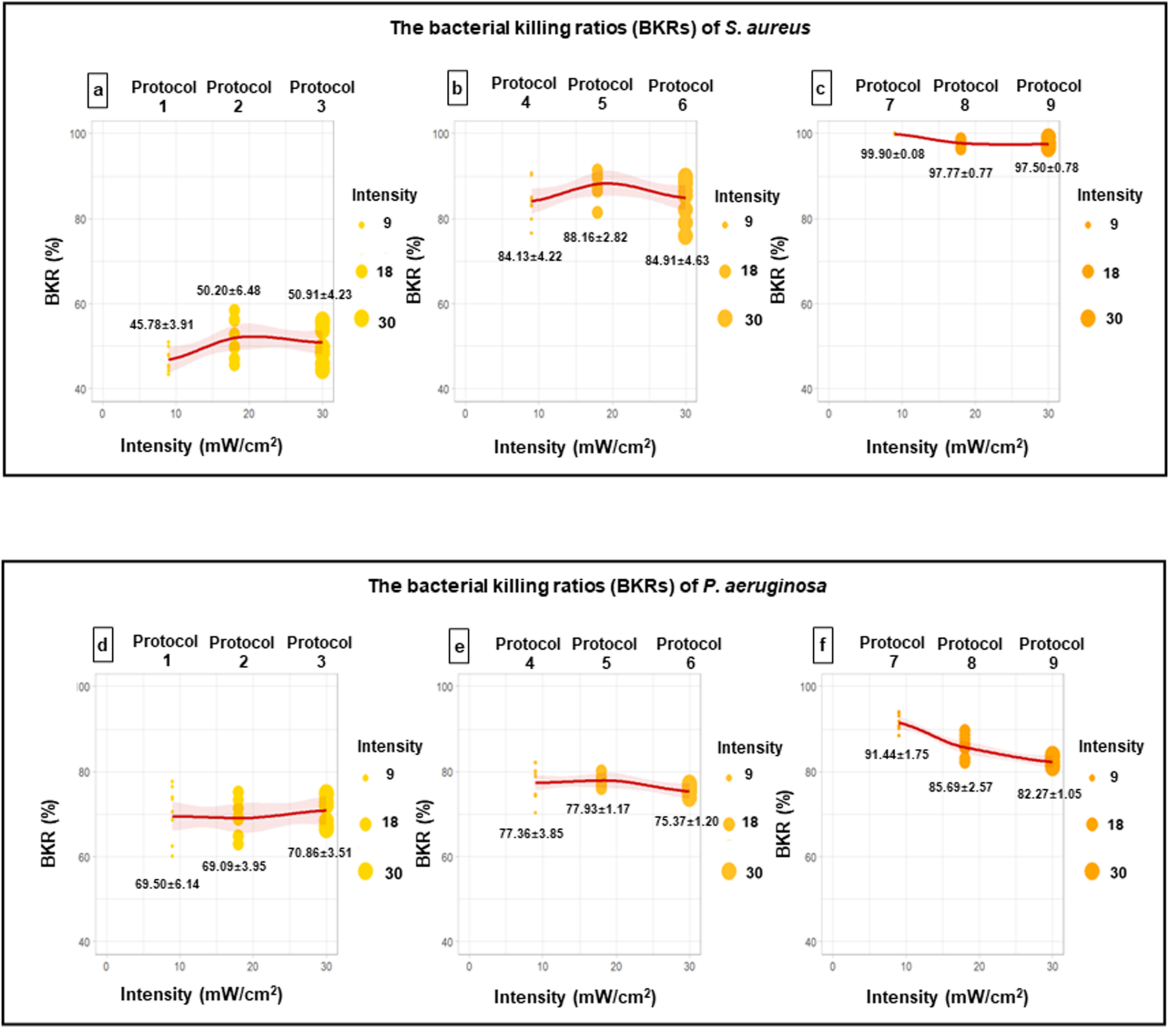
The BKRs of *S. aureus* (a, b, c) and *P. aeruginosa* (d, e, f) under the same total fluence of 5.4 J/cm^2^ (protocols 1–3), 10.0 J/cm^2^ (protocols 4–6), and 15.0 J/cm^2^ (protocols 7–9) with different irradiation intensity.

As shown in Figures 1a, 1b, 1d, and 1e, a shorter irradiation time maintaining a total fluence of 5.4 or 10.0 J/cm^2^ did not seem to have a negative effect on the BKR (*P* = 0.106, 0.120, 0.284, and 0.105, respectively). In the 15.0 J/cm^2^ total fluence protocols (protocols 7–9), increasing acceleration (higher UV intensity, shorter duration) resulted in a statistically significant decrease of BKR in both *S. aureus* (protocol 7 vs. protocols 8 and 9 [both *p* < 0.001]; protocol 8 vs. protocol 9 [*P* = 0.404]) (Fig. 1c) and *P. aeruginosa* (protocol 7 vs. protocols 8 and 9, protocol 8 vs. protocol 9; all *P* < 0.001) (Fig. 1f).

**Figure 2.**
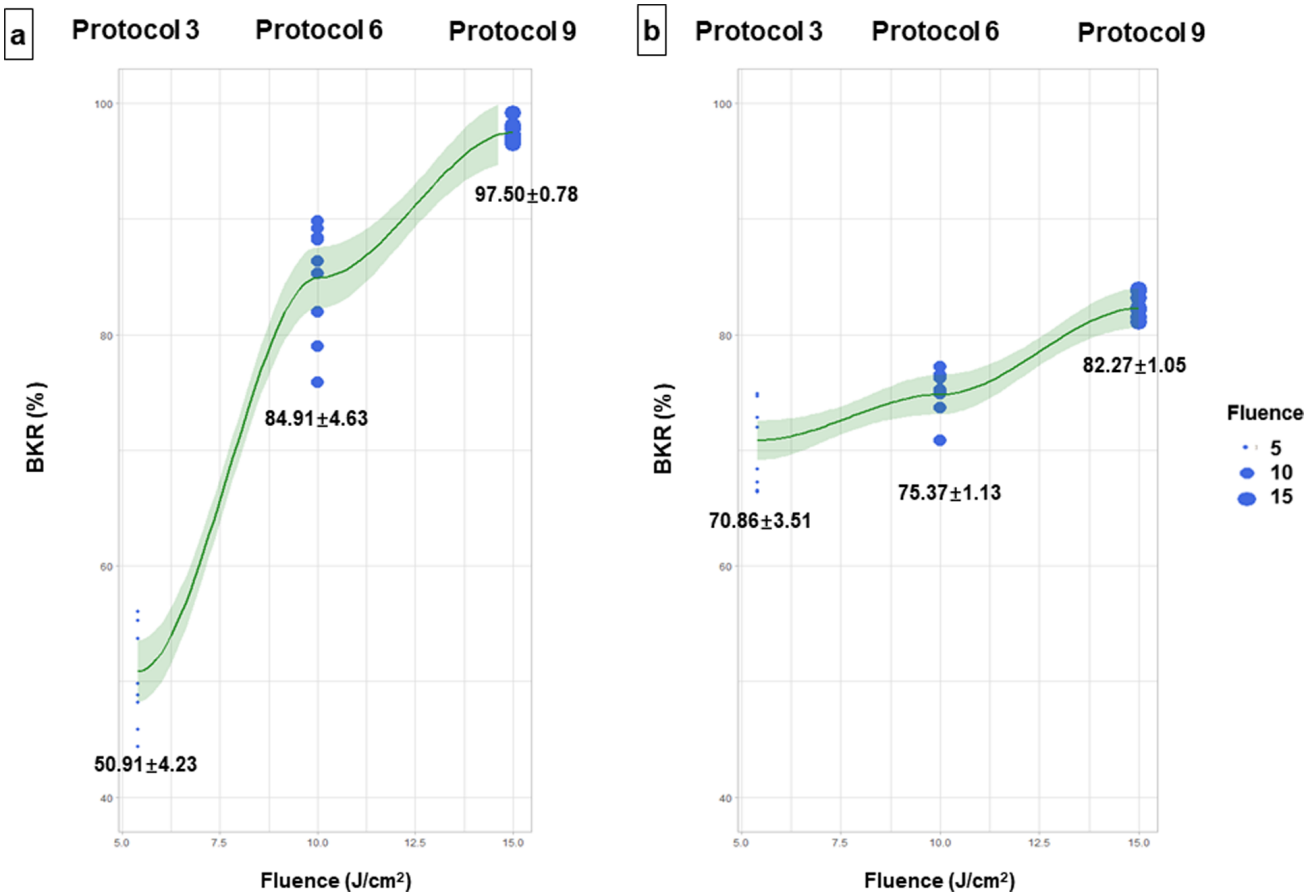
The BKRs of *S. aureus* **(a)** and *P. aeruginosa* **(b)** under the same irradiation intensity (30 mW/cm^2^) with three different total fluences (protocol 3, protocol 6, and protocol 9).

Using an irradiation intensity of 30 mW/cm^2^ with fluences of 5.4 (protocol 3), 10.0 (protocol 6), and 15.0 J/cm^2^ (protocol 9), the mean BKR of *S. aureus* was 50.91%, 84.91%, and 97.50%, respectively (Fig. 2a), whereas the mean BKR of *P. aeruginosa* was 70.86%, 75.34%, and 82.27%, respectively (Fig. 2b). The statistical analyses showed that the increase in BKR was significant with increasing fluences (all *P* < 0.001).

### Comparisons of PACK-CXL–Induced BKRs Under Protocol 9

The average PACK-CXL–induced BKR was 97.50% for *S. aureus*, compared with 82.27%, 41.97%, 65.38%, and 78.04% for *P. aeruginosa*, *A. xylosoxidans*, *S. epidermidis,* and *S. maltophilia*, respectively. Across all strains, a statistical difference was found between every two strains (all *P* < 0.001).

## Discussion

This in vitro study tried to prove that, besides the bactericidal effect of UV-A light itself, the bacterial killing effect induced by accelerated high-fluence PACK-CXL could effectively decrease the bacterial concentration of several clinically significant bacterial strains obtained from the clinical corneal infection samples, while the Bunsen–Roscoe law of reciprocity was still simultaneously followed at the relatively high total fluence level.

For the bacterial strains investigated in the current study, in bacterial keratitis and more specifically in contact lens-associated keratitis, *S. aureus* represents the most common gram-positive organism*,* and *P. aeruginosa* is the most common gram-negative organism. Both strains might rapidly lead to vision-threatening keratitis.^14^^,^^15^ In addition, *S. epidermidis* is one of the most commonly implicated pathogens in polymicrobial keratitis.^14^ The other strains (*A. xylosoxidan*s and *S. maltophilia*) investigated here were chosen as the references for emerging, multidrug-resistant pathogens.^16^^–^^18^

In the current study, to objectively investigate the bacterial killing effect induced by PACK-CXL, we have first proven that the chromophore itself—riboflavin—did not influence bacterial growth. We also noticed that, except for *S. epidermidis*, the number of the CFUs in all other bacterial strains without PACK-CXL (in group A and group B) was close to 10^3^ in the final diluted solutions. This finding may be explained by the fact that some strains, especially gram-positive bacteria, form strong clusters that have an influence on turbidity measurements.^19^ Therefore, it seems to be true for this certain *S. epidermidis* strain, where the McFarland 0.5 solution reached only approximately 0.25 × 10^8^ CFU/mL. Then, the UV-A light irradiation was applied in the bacteria solution that contains riboflavin (group D) or not (group C) to directly confirm that these bacterial killing effects were not induced by UV-A light only and the PACK-CXL–induced bacterial killing effects were relevant (Table 2). Interestingly, neither gram status (gram positive: *S. aureus* and *S. epidermidis*) nor the aerobic/anaerobic state of the bacterial strains seemed to be predictive of PACK-CXL BKR efficacy, postulating rather a protective effect due to the properties of the bacterial cell wall, for example, molecular composition, thickness, or charge. This composition is often not only bacterial species dependent, but might even be different between strains of the same species.^20^

For the PACK-CXL protocol settings, as per our previous study findings,^13^ we once again found that, when higher total fluence PACK-CXL protocols were applied in all bacterial strains, the BKR would correspondingly increase. One potential concern might be if the higher total fluence PACK-CXL protocols were applied in clinical practice, the high fluence might potentially risk causing damage to the corneal endothelium. However, for CXL in transparent keratoconic and myopic corneas, previous studies have investigated the safety of the endothelium,^21^^,^^22^ showing that accelerated CXL protocols with total fluences of up to 15 J/cm^2^ did not affect endothelial cell density. Similarly, Seiler et al.^23^ have recently shown that the damage threshold of the corneal endothelium in transparent corneas might be substantially higher than previously anticipated. Unlike keratoconic and myopic corneas, corneas with bacterial keratitis usually display edema and are opaque, decreasing UV-A transmission through the stroma. This factor should further lower the potential risk of endothelial cell damage caused by PACK-CXL with a total fluence of 15 J/cm^2^.^24^

The Bunsen–Roscoe law of reciprocity states that the same photochemical effect can be achieved with a shorter irradiation time and increased radiation intensity if the total dose remains the same.^25^ Originating from photochemistry, this theoretical law should not be used steadily in biological systems. In the context of CXL research, however, this law has been discussed numerous times in publications over the past decade.^26^ In the current study, up to a total fluence of 10 J/cm^2^, lower irradiation intensity protocols (protocols 1 and 4) achieved similar *S. aureus* and *P. aeruginosa* BKRs as higher irradiation intensity protocols (protocols 2 and 3 vs. protocol 1; protocols 6 and 7 vs. protocol 4). However, at total fluences of 15 J/cm^2^, when both *S. aureus* and *P. aeruginosa* were irradiated with higher intensities in a shorter time (protocols 8 and 9), a slight decrease in BKR was observed, relative to the low-intensity protocol (protocol 7), indicating that the Bunsen–Roscoe law was not respected fully. Nevertheless, the BKR remained highly satisfactory as it exceeded 95% in *S. aureus* and 80% in *P. aeruginosa*. Moreover, faster irradiation protocols are more easily applied in clinical practice, especially when performed in an office-based slit lamp procedure.^27^ Such an approach can allow ophthalmologists to perform a timely intervention on the diagnosis of bacterial keratitis, which may improve patients' prognosis, relative to the normal practice of arranging the bacterial keratitis patients to be at the end of the operating room schedule to avoid introducing the pathogen to the operating room and, thus, delaying treatments.

The slight decrease of BKR with the 15.0 J/cm^2^ total fluence accelerated protocols might be due to some potential rate-limiting effects that we considered: (1) the lower rate at which oxygen diffuses from the atmosphere into the reaction well plates, which may have reduced the rate of the UV-A riboflavin photo-oxidative reaction^28^; (2) the bacteria are able to survive reactive oxygen species for a short while, meaning PACK-CXL treatment is too short to reach a linear kill curve; and (3) radicals are not produced fast enough in the accelerated PACK-CXL procedures. We know from CXL for ectasia that the corneal biomechanical stiffing effect decreases significantly when 5.4 J/cm^2^ total fluence UV irradiation protocols are accelerated (18 mW/cm^2^),^29^ whereas accelerated PACK-CXL–induced bacterial killing effects are similar across fluences up to 10.0 J/cm^2^ and remain at promising levels up to at least 15.0 J/cm^2^ fluence.

It is worth noting in this study that the chosen amount of bacteria in the wells of the microtiter plate differs from the amount present in infected corneas. However, the choice to irradiate a volume of 11 µL in a well of a 96-well plate corresponds, as previously described, to a corneal thickness of around 285 µm, which is within the known CXL penetration depth of approximately 300 µm.^13^ Moreover, we chose a very high initial bacterial load (10^7^/mL). As shown by Badenoch et al.,^30^ in a rat model for bacterial keratitis, the maximum bacterial count of 10^7^ was found 48 hours after infection.

The in vitro setting is a limitation of this study, because it neither considers the role of the extracellular matrix nor the eventual immune response. At the same time, the presence of an immune response and the concomitant use of antibiotics might enhance the positive effect of PACK-CXL. The ex vivo study is indicated for us to validate the results of the current study. In addition, further experiments are required to investigate the safety of high-fluence PACK-CXL in infected corneas and the actual role of oxygen in PACK-CXL.

In conclusion, our study showed that high-fluence accelerated PACK-CXL is effective in decreasing the bacterial concentration in vitro in several of the strains that are most commonly responsible for keratitis, and that PACK-CXL is associated with different BKRs depending on the bacterial strain. With regard to the clinical setting, high-fluence accelerated PACK-CXL holds the potential to significantly improve infectious keratitis treatment outcomes. Further research is needed to optimize the specific PACK-CXL protocol for distinct bacterial strains.
